# Treatment of very high risk membranous nephropathy complicated by pulmonary embolism with glucocorticoids and rituximab: Case report

**DOI:** 10.1097/MD.0000000000041241

**Published:** 2025-01-10

**Authors:** Umer Farooq Khan, Charmaine Sia, Thomas Paulraj Thamboo, Horng Ruey Chua, Gek Cher Chan

**Affiliations:** a Division of Nephrology, Department of Medicine, National University Hospital, Singapore; b Department of Pathology, National University Hospital, Singapore; c Department of Medicine, Yong Loo Lin School of Medicine, National University of Singapore, Singapore.

**Keywords:** Anti-phospholipase 2 receptor antibody, Membranous nephropathy, rituximab, thromboembolism

## Abstract

**Rationale:**

We report the efficacy of combination prednisolone and intravenous (IV) rituximab as an immunosuppressive regimen for a young male presenting with extensive venous thromboembolism including a submassive pulmonary embolism secondary to life-threatening nephrotic syndrome from very high risk anti-phospholipase-A2 receptor (PLA2R) positive membranous nephropathy. Initial treatment was with mechanical thrombectomy and anticoagulation. Thereafter, oral prednisolone was initiated to induce remission, during a period of uninterrupted anticoagulation. He subsequently underwent a kidney biopsy for histological confirmation and IV rituximab was administered as definitive treatment.

**Patient concerns:**

A 33-year-old Chinese male with no chronic conditions presented with shortness of breath and left-sided pleuritic chest pain.

**Diagnoses:**

He was diagnosed with a submassive pulmonary embolism which was confirmed on computer tomography imaging with additional extensive clot burden in the inferior vena cava and renal veins. Further workup revealed nephrotic syndrome, with proteinuria of 6.5g/day, and serum Albumin 26 g/L, associated with anti-PLA2R of 223 RU/ml. His presenting serum creatinine was 108 µmol/L (CKD-EPI eGFR 77ml/min/1.73m^2^). Additional workup for malignancy and infections were negative.

**Interventions:**

As part of acute management, immediate anticoagulation was initiated. The patient then underwent endovascular thrombectomy and inferior vena cava filter placement. Given the emergent indication for and need for 4 weeks of uninterrupted anticoagulation, his kidney biopsy had to be delayed. The patient was then preemptively treated with IV Methylprednisolone 500mg for 3 days followed by 0.5mg/kg of oral prednisolone after taking into consideration the specificity of PLA2R positivity for membranous nephrology.

**Outcomes:**

After 4 weeks of treatment, serum albumin improved to 32 g/L and anti-PLA2R levels improved significantly to 27 RU/ml. His subsequent kidney biopsy confirmed membranous nephropathy and 2 doses of IV rituximab 1g were administered 14 days apart. Six months after initial presentation, the patient is in partial remission. Albumin has improved to 41 g/L, Anti PLA2R < 2 RU/ml, and proteinuria is 1.18g/day.

**Lessons:**

This case demonstrates that preemptive treatment in patients with anti-PLA2R positive membranous nephropathy can initiated without a histological diagnosis when there are strong contraindications against a kidney biopsy. Treatment with a combination of steroids and IV rituximab could be a viable treatment option for patients with very high-risk membranous nephropathy over conventional therapy with cyclophosphamide.

## 1. Introduction

Membranous nephropathy (MN) is characterized by autoantibodies targeting podocyte antigens, such as Anti-phospholipase-A2 receptor (PLA2R) or Anti-Thrombospondin Type 1 (THSD7A), while 20% to 30% are secondary to immune complex deposition due to an underlying condition.^[1]^ Therefore, clinical assessment is essential to evaluate for malignancy, infections, autoimmune conditions, or drug-related triggers. Spontaneous remission occurs in 40% of cases, while 20% progress to kidney failure.^[1]^ Venous thromboembolic (VTE) complications in nephrotic syndrome arise from urinary loss of antithrombin III and reduced protein S activity.^[2]^ Clinically evident VTE events affect 7% of MN patients,^[3]^ and some studies suggest that Anti-PLA2R-associated MN carries a higher VTE risk.^[4]^

This report presents a case of a young male with extensive VTE including a submassive pulmonary embolism due to life-threatening nephrotic syndrome from very high risk anti-PLA2R-positive MN, discussing treatment strategies whena kidney biopsy is deferred due to anticoagulation risks. We also report the efficacy of combination steroid and rituximab treatment which presents as an alternative over conventional oral cyclophosphamide in very high risk MN, mitigating the undesired complications of alkylating therapy.

## 2. Case presentation

A 33-year-old Chinese driver with no medical history presented with a 2-month history of cough, left-sided pleuritic chest pain, and worsening dyspnea. He was an active smoker (10 pack-years) and consumed alcohol twice weekly. Previously, he was treated for community-acquired pneumonia with Augmentin and Azithromycin. He had also been referred to the local tuberculosis unit, where sputum acid-fast bacilli smear, and molecular detection of tuberculosis were negative. He denied hemoptysis, purulent sputum, night sweats, palpitations, or dizziness, but noted a 3kg weight loss over 2 months. A review of systems was negative for rash, photosensitivity, or joint pain.

On examination, he was afebrile, with a blood pressure of 123/97mmHg, heart rate of 122 bpm, and oxygen saturation of 97% on room air. Respiratory examination revealed reduced breath sounds at the right base. A chest X-ray showed a new right lower zone opacity, and he was treated with Augmentin and azithromycin.

**Figure 1.**
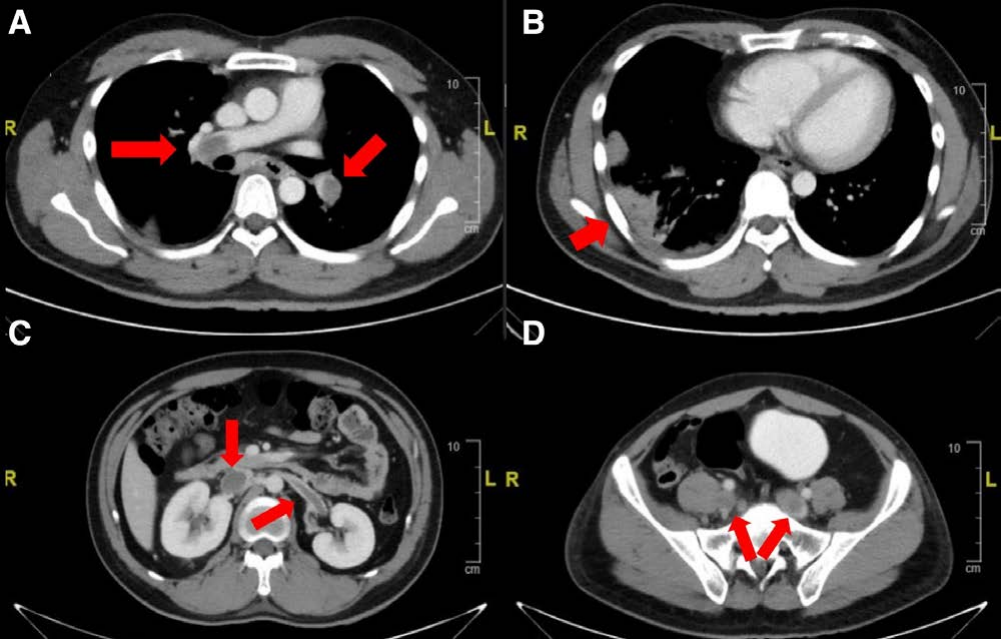
(A) Bilateral pulmonary emboli in main pulmonary trunks. (B) Wedge-shaped infarct in right lower lobe. (C) Bilateral renal vein thrombosis. (D) Bilateral iliac vein thrombosis

Initial investigations showed leucocytosis of 13.6 × 10^9^/L, while hemoglobin and platelets were normal. His serum creatinine was 108 *μ*mol/L (Chronic Kidney Disease Epidemiology Collaboration (CKD-EPI) eGFR 77 ml/min/1.73m^2^).^[5]^ A computer tomography (CT) scan of his chest showed bilateral pulmonary emboli in both main pulmonary arteries and distal branches, with a wedge-shaped infarct and necrosis in the right lower lobe. The thrombus extended to the inferior vena cava (IVC), bilateral renal veins, and iliac veins (Fig. 1). Anticoagulation was immediately initiated with subcutaneous Enoxaparin. An echocardiogram demonstrated right ventricular dysfunction characterised by a dilated right ventricle with mild systolic dysfunction, pulmonary hypertension, and mild tricuspid regurgitation. Patient’s care was transferred to a tertiary hospital for further management.

Further workup confirmed nephrotic syndrome with 24-hour urinary protein at 5.6g/day and serum albumin of 26 g/L. Urine microscopy revealed 26 red blood cells per high-power field. Anti-PLA2R antibody titers were elevated at 223 RU/ml, while markers such as Anti-THSD7A, complements, and dsDNA were negative, confirming MN. The patient also had hyperlipidemia, with LDL at 4.1 mmol/L and total cholesterol at 7.0 mmol/L.

**Figure 2.**
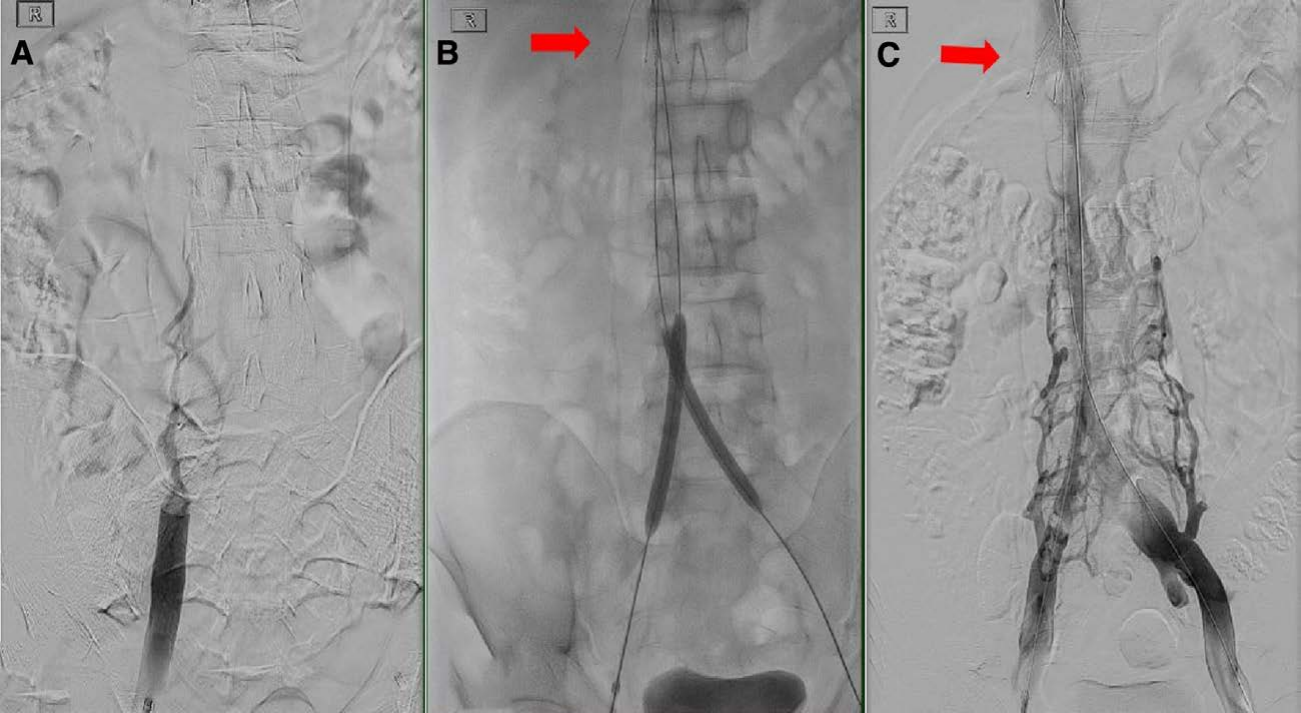
IVC filter was inserted via right internal jugular access prior to procedure (arrow). (A) Initial angiogram showing non-opacification of IVC from right femoral vein access. (B) Bilateral iliac vein angioplasty after angio-jet thrombolysis. (C) Restoration of blood flow with collaterals in IVC.

A repeat CT scan after 1 week of anticoagulation showed stable findings, leading to the insertion of an IVC filter on day 11. Thrombectomy and thrombolysis with catheter-administered rTPA, angio-jet thrombectomy, and balloon angioplasty were performed on the iliac veins and IVC (Fig. 2), achieving partial recanalization. Warfarin was then initiated to maintain an INR of 2.0 to 3.0.

A kidney biopsy to confirm MN was deferred due to the need for 4 weeks of uninterrupted anticoagulation. Given the urgency of inducing remission in a patient with severe thromboembolic disease and elevated Anti-PLA2R levels, immunosuppression was started without a biopsy. After screening for malignancy and infection, on Day 13, IV Methylprednisolone 500mg daily was administered for 3 days, followed by oral prednisolone 35mg daily (0.5mg/kg/day). Serum creatinine remained stable at 100 µmol/L (CKD-EPI eGFR 85ml/min/1.73m²).

Due to concerns of hospital-acquired pneumonia and blurred vision 2 days after starting steroids, the prednisolone dose was reduced to 20mg daily. Losartan 25mg daily was initiated for blood pressure control and antiproteinuric effect. He also received Vitamin D supplementation and nebulized pentamidine for *Pneumocystis jirovecii pneumonia* (PJP) prophylaxis. After 4 weeks of uninterrupted anticoagulation, the patient was re-admitted for a kidney biopsy with intravenous heparin bridging. He remained clinically stable and resumed anticoagulation safely postprocedure.

**Figure 3.**
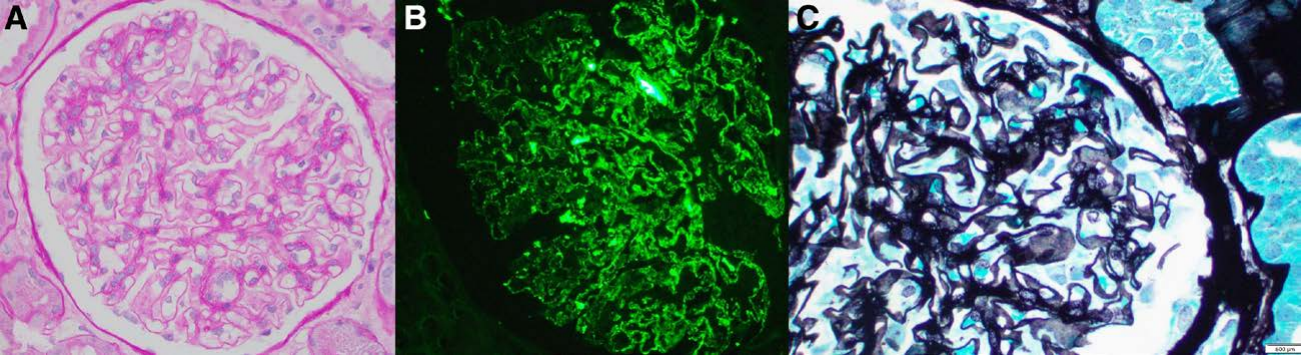
(A) Glomerulus with rigid-appearing capillary loops, with absence of endocapillary hypercellularity (periodic acid-Schiff stain, original magnification: 400×). (B) The glomerular capillary loops show “holes” and/or “spikes” on the PAAG stain (periodic acid-silver (PAAG) stain, original magnification 600×). (C) Immunofluorescence staining for PLA2R showing moderate (2 + intensity) finely to coarsely granular capillary loop staining (original magnification: 400×).

Histopathological examination confirmed the diagnosis of MN (Fig. 3). The glomeruli had rigid-appearing capillary loops on the periodic acid-Schiff stain, which showed “holes” and “spikes” on the periodic acid-silver (PAAG) stain. There was no endocapillary hypercellularity or segmental sclerosis. On immunofluorescence staining, there was strong (3 + intensity) finely to coarsely granular glomerular capillary loop staining for IgG and Kappa light chain, and moderate (2 + intensity) finely to coarsely granular glomerular capillary loop staining for PLA2R and Lambda light chain. Electron microscopy showed numerous subepithelial electron-dense deposits of varying sizes, along with diffuse podocyte foot process effacement.

By week 6, after 4 weeks on Prednisolone 20mg daily, serum albumin improved to 32g/L, and anti-PLA2R reduced to 27 RU/ml, though 24-hour urine protein remained high at 5.8g/day. The risks and benefits of the modified Ponticelli regimen (cyclophosphamide) versus rituximab were discussed. Given his age, desire to maintain fertility, and concerns about drug toxicity, the patient chose rituximab. He received 2 IV doses of rituximab 1g, 2 weeks apart (weeks 12 and 14). CD19 and CD20 subsets were monitored to confirm B-lymphocyte suppression.

A follow-up CT scan 3 months after his initial presentation showed significantly reduced clot burden, with only a small residual clot in the left lower lung lobe. The IVC filter was removed, and Warfarin was continued. Six months since the original presentation (three months post-IV rituximab), the patient remains in partial remission. Anti-PLA2R (<2 RU/ml) and serum albumin (41g/L) levels normalized, and proteinuria decreased to 1.18g/day. He is on a tapering prednisolone dose (currently 5mg daily), Losartan 100mg daily, and co-trimoxazole for PJP prophylaxis, with stable liver function tests.

## 3. Discussion

This case highlights the challenges of managing severe, life-threatening VTE complications in nephrotic syndrome, and the importance preemptive treatment to gain disease control and prevent further complications while facilitating definitive diagnosis to subsequently tailor treatment to achieve remission. The KDIGO 2021^[6]^ guidelines recommend using anti-PLA2R antibodies for diagnosing MN but also suggest kidney biopsy before initiating immunosuppression. The predicament is this case, is the timing of the kidney biopsy. Though indicated, should there be significant bleeding post biopsy, anticoagulation must be interrupted for a prolonged period which would exacerbate the thrombotic burden. However, delaying tissue diagnosis can hinder definitive diagnosis and treatment. For anti-PLA2R-negative MN or other nephrotic syndrome etiologies, clinicians will face the dilemma of performing an early high-risk biopsy or proceeding with empirical immunosuppressive therapy if anticoagulation cannot be interrupted.

The KDIGO Guidelines also recommend alternating glucocorticoids and cyclophosphamide in severe or life-threatening MN due to limited data on rituximab in such cases and the high mortality rates from uncontrolled VTE. Retrospective studies have shown remission with rituximab in patients with advanced CKD with eGFR as low as 18 ml/min/1.73m^2^.^[7,8]^ Despite potential concerns about rituximab’s delayed onset, we opted for it over cyclophosphamide given the patient’s preferences and fertility considerations. Glucocorticoids were used as bridging therapy to induce rapid remission and reduce thromboembolic risk.

## 4. Conclusions

To our knowledge, this is the first reported case of successful remission in a patient with severe MN and life-threatening VTE using steroids and rituximab. This regimen may be considered in patients who wish to avoid complications from cyclophosphamide. However, further prospective studies are required to evaluate long-term outcomes.

Declarations

**Ethics approval and consent to participate:** The authors have no ethical conflicts to disclose. The subject has given written informed consent to publish his case including publication of images. This case study was conducted ethically in accordance with the World Medical Association Declaration of Helsinki.

**Availability of data and materials:** Data sharing not applicable to this article as no datasets were generated or analysed during the current study.

**Competing interests:** The authors have no relevant financial or non-financial interests to disclose.

**Funding:** The authors received no specific funding for this work.

## Author contributions

**Conceptualization:** Umer Farooq Khan.

**Supervision:** Horng Ruey Chua, Gek Cher Chan.

**Visualization:** Thomas Paulraj Thamboo.

**Writing – original draft:** Umer Farooq Khan, Charmaine Sia.

**Writing – review & editing:** Umer Farooq Khan, Charmaine Sia, Thomas Paulraj Thamboo, Horng Ruey Chua, Gek Cher Chan.
